# Loss of KAP3 decreases intercellular adhesion and impairs intracellular transport of laminin in signet ring cell carcinoma of the stomach

**DOI:** 10.1038/s41598-022-08904-8

**Published:** 2022-03-23

**Authors:** Tomohiro Soda, Yasuyuki Gen, Kei Terasaki, Naoto Iwai, Tomoko Kitaichi, Osamu Dohi, Hiroyoshi Taketani, Yuya Seko, Atsushi Umemura, Taichiro Nishikawa, Kanji Yamaguchi, Michihisa Moriguchi, Hideyuki Konishi, Yuji Naito, Yoshito Itoh, Kohichiroh Yasui

**Affiliations:** 1 Department of Molecular Gastroenterology and Hepatology, Kyoto Prefectural University of Medicine, Kamigyo, Kyoto 602-8566 Japan; 2 School of Health Sciences, Bukkyo University, Nakagyo, Kyoto 604-8418 Japan

**Keywords:** Cancer, Cell biology, Molecular biology, Gastroenterology, Oncology, Pathogenesis

## Abstract

Signet-ring cell carcinoma (SRCC) is a unique subtype of gastric cancer that is impaired for cell–cell adhesion. The pathogenesis of SRCC remains unclear. Here, we show that expression of kinesin-associated protein 3 (KAP3), a cargo adaptor subunit of the kinesin superfamily protein 3 (KIF3), a motor protein, is specifically decreased in SRCC of the stomach. CRISPR/Cas9-mediated gene knockout experiments indicated that loss of KAP3 impairs the formation of circumferential actomyosin cables by inactivating RhoA, leading to the weakening of cell–cell adhesion. Furthermore, in *KAP3* knockout cells, post-Golgi transport of laminin, a key component of the basement membrane, was inhibited, resulting in impaired basement membrane formation. Together, these findings uncover a potential role for KAP3 in the pathogenesis of SRCC of the stomach.

## Introduction

Gastric cancer (GC) is one of the most common causes of cancer mortality worldwide. The great majority of GCs are adenocarcinomas, which are classified histologically into intestinal and diffuse types based on Lauren’s classification^1^. Intestinal-type GC usually forms tubular or glandular structures and often arises through atrophic gastritis and intestinal metaplasia caused by long-lasting *Helicobacter pylori* infection. In contrast, diffuse-type GC exhibits a lack of cell cohesion, such that individual cells infiltrate and thicken the stomach wall without forming a discrete mass; diffuse-type GC often arises from *Helicobacter pylori*-negative gastric mucosa^2^. Diffuse-type GC typically occurs in younger patients and can develop throughout the stomach; this type of GC can result in a loss of distensibility of the gastric wall (so-called linitis plastica), and carries a poor prognosis^3^.

According to the World Health Organization (WHO)’s classification of GC, the Lauren’s diffuse type corresponds to poorly cohesive carcinomas^4^. Signet-ring cell carcinoma (SRCC) is defined as a poorly cohesive carcinoma composed predominantly of tumor cells with ample cytoplasmic mucin that appears optically clear on hematoxylin and eosin (H&E) staining and an eccentrically positioned nucleus. In the Japanese classification of GC, Lauren’s intestinal type is subdivided into papillary, tubular, and mucinous adenocarcinomas, and the diffuse type is subdivided into SRCC and poorly differentiated adenocarcinoma^5^.

SRCC has a unique epidemiology among GCs. Despite recent decreases in the overall incidence and mortality of GC, the incidence of SRCC has continued to increase^6^, accounting for more than 30% of gastric adenocarcinoma in recent studies^7,8^. SRCC is associated with more-advanced tumor stage and is more likely to have lymph node spread and distant metastases than other histological subtypes of GC^9^. In advanced SRCC, peritoneal carcinomatosis frequently is observed^10^. Although SRCC is positively related to survival outcomes in early tumor stages, SRCC paradoxically is associated with worse prognosis and lower chemosensitivity compared to non-SRCC at advanced tumor stage^11^.

One of the characteristic pathological findings of SRCC is loss of cell–cell adhesion. E-cadherin, which is encoded by *CDH1*, is a key molecule of cell–cell adhesion, binding epithelial cells together through adherens junctions. *CDH1*’s role in carcinogenesis and epithelial-mesenchymal transition has been well investigated in many types of cancers. *CDH1* germline mutations account for the main genetic causes of hereditary diffuse-type GC^12^. Loss-of-function mutations in the gene encoding E-cadherin also have been observed in sporadic SRCC and may be involved in initiation of SRCC^13^.

SRCC has a specific oncogenesis. Whole-genome sequencing has revealed that SRCC tumors often harbor mutations in any of six genes, including *TP53*, *CDH1*, *PIK3CA*, *ERBB2*, *LCE1F*, and *OR8J1*^14^. However, genes that have been reported to be frequently mutated in diffuse-type GC, such as *ARID1A*, *RHOA*, and *SMAD4*, are not significantly mutated in SRCC, indicating possible distinct genomic features of SRCC compared to non-SRCC diffuse-type GC. Although *RHOA* is rarely mutated in SRCC, mutations often are seen in genes encoding regulators of RhoA such as RhoGAPs (GTPase-activating proteins) or RhoGEFs (GDP/GTP-exchange factors); additionally, a *CLDN18*-*ARHGAP26/6* fusion frequently is present in SRCC. *CLDN18* encodes a component of tight junctions, while *ARHGAP26* encodes an inhibitor of RhoA. The hybrid protein produced by this gene fusion has been shown to reduce cell–cell and cell-extracellular matrix (ECM) adhesion^15^. Furthermore, protein–protein interaction network analyses of SRCC have identified mutations in over a hundred other genes encoding cell adhesion-related proteins, demonstrating the importance of cell adhesion in SRCC tumorigenesis^14^. However, the molecular mechanism of the loss of cell–cell adhesion in SRCC remains poorly understood.

Normal epithelial cells are stably connected to each other via the apical junctional complex (AJC), which consists of tight junctions and adherens junctions^16^. The AJC associates with circumferential actomyosin cables, and contraction of these cables produces tension over the AJC. This force is important for defining epithelial architecture. Actomyosin contraction is governed by the RhoA signaling pathway and RhoA’s downstream effector mDia1 (the mammalian homologue of the *Drosophila* diaphanous 1 protein)^17^. However, AJCs tend to be disrupted during tumor progression, and this disruption has been implicated in cancer dissemination.

Cell polarization and the formation of cell–cell junctions are coupled processes that are essential to tissue morphogenesis. The ECM represents a potential link between polarity and tissue organization^18^. Dynamic cell–ECM interactions are integral to tissue morphogenesis, as illustrated by the reciprocal relationship between epithelia and their laminin-rich basement membrane. In developing epithelia, cells secrete laminin and assemble the protein into a polymeric network through the activity of cell-surface laminin receptors^19^. Loss of cell–cell adhesion and cell polarity is commonly observed in advanced cancers and correlates strongly with cancer invasion into adjacent tissues through the basement membrane and ECM.

Intracellular transport driven by motor proteins is essential for cellular function and morphogenesis. Kinesin superfamily proteins (KIFs) are motor proteins that transport membranous organelles and macromolecules fundamental for cellular functions along microtubules. The kinesin superfamily protein 3 (KIF3), a KIF member, is a heterotrimeric complex that consists of two kinesin motor proteins, KIF3A and KIF3B, and a cargo adaptor subunit, KAP3^20^.

In the present study, we sought to elucidate the molecular mechanism of SRCC development. We investigated the expression profile of genes potentially related to cell–cell adhesion in GC, and found that expression of *KAP3* (also known as *KIFAP3*) is decreased in SRCC. Using CRISPR/Cas9-mediated gene knockout of *KAP3*, we provided evidence that loss of *KAP3* may be involved in the carcinogenesis of SRCC of the stomach by weakening RhoA-mediated cell–cell adhesion and impairing basement membrane formation.

## Materials and methods

### Antibodies

Antibodies against KAP3 (Cat. sc-55598), β-actin (Cat. sc-47778), E-cadherin (Cat. sc-8426) and β-catenin (Cat. sc-1496) were purchased from Santa Cruz Biotechnology (Dallas, TX, USA). Antibodies against laminin (Cat. Ab11575) and giantin (Cat. ab37266) were obtained from abcam (Cambridge, MA, USA). The antibody against ZO-1 (Cat. #61-7300) was obtained from Invitrogen (Carlsbad, CA, USA). The antibody against RhoA (Cat. #ARH05) was obtained from Cytoskeleton (Denver, CO, USA).

### Cell lines

Six human gastric cancer cell lines (MKN7, MNK74, MNK45, NUGC3, NUGC4, and KATOIII) were obtained from the JCRB Cell Bank (Osaka, Japan) and the RIKEN Bioresource Center (Tsukuba, Japan). All cell lines were cultured in RPMI 1640 supplemented with 10% fetal bovine serum at 37 °C in a humidified atmosphere containing 5% CO_2_.

### Generation of MKN74 cell line harboring *KAP3* gene knockout by CRISPR/Cas9 system

*KAP3* gene knockout (KO) was performed using the Alt-R CRISPR-Cas9 System (Integrated DNA Technologies (IDT), Coralville, IA, USA), according to the manufacturer's protocol. A predesigned guide RNA (gRNA) was purchased from IDT. The target sequence of the gRNA was GGACCCTTGCTAATGCACCA, which was followed by a protospacer adjacent motif (PAM) sequence, AGG. The ribonucleoprotein comprising Cas9 protein and gRNA was delivered into MKN74 cells using Lipofectamine RNAiMAX (Invitrogen) according to the manufacturer's protocol. After transfection, MKN74 cells were seeded in 35-mm dishes at a density of 500 cells/dish. Single-cell colonies were recovered and expanded. Clones were tested for lack of KAP3 protein expression using immunoblotting analysis. One clone that had been subjected to genome editing and shown to lack KAP3 expression was selected for further experiments.

### Forced expression of the *KAP3* gene

A full-length human *KAP3* expression vector constructed with a HaloTag cDNA (FHC20706E) was obtained from Promega (Madison, WI, USA). The vector was transfected into cells using Lipofectamine 3000 (Invitrogen). The HaloTag Oregon Green ligand (Promega) was used to detect HaloTag-fused KAP3 protein.

### Quantitative reverse transcription-PCR (qRT-PCR)

Total RNA was extracted from each cell line using TRIzol (Thermo Fisher Scientific, Waltham, MA, USA) according to the manufacturer’s protocol. Residual genomic DNA was removed by incubating the RNA samples with RNase-free DNase I (Takara Bio, Otsu, Japan) prior to performing qRT-PCR. The mRNA levels were quantified by qRT-PCR using the LightCycler 96 system (Roche Diagnostics, Mannheim, Germany) and the KAPA SYBR FAST Universal Kit (KAPA Biosystems, Cape Town, South Africa) according to the manufacturer's protocol. The endogenous control for mRNA was the *ACTB* transcript encoding the housekeeping protein β-actin. The primers used were as follows: *KAP3* (*KIFAP3*) mRNA: forward, AGGAGCCATAAGTCCCGATT, and reverse, GTCCAAGAATGCCAACTGGT; *RHOA* mRNA: forward, AAGGACCAGTTCCCAGAGGT, and reverse, GCTTTCCATCCACCTCGATA; *ACTB* mRNA: forward, GTCCACCTTCCAGCAGATGT, and reverse, TGTTTTCTGCGCAAGTTAGG.

### Immunoblotting

Immunoblotting was performed as described previously^21^. The anti-KAP3, anti-laminin, and anti-β-actin antibodies were used for immunoblotting at dilutions of 1:500, 1:400, and 1:2000, respectively. For immunodetection, anti-rabbit IgG or anti-mouse IgG antibody (Cell Signaling Technology, Beverly, MA, USA) was used as the secondary antibody at a dilution of 1:5000 or 1:10,000, respectively. Antibody binding was detected using the Enhanced ChemiLuminescence (ECL) system (GE Healthcare, Chicago, IL, USA).

### Immunohistochemistry

Immunohistochemistry was performed as described previously^22^. The anti-KAP3 antibody was used for immunohistochemistry at a dilution of 1:50. The GC tissue microarray (Cat. #NBP2-30308) was obtained from Novus Biologicals (Centennial, CO, USA). Expression levels were evaluated semi-quantitatively as follows: each sample was scored for two parameters. The first parameter was the percentage of positive cells, which was determined using a scale as follows: 0, absence of positive cells; 1, < 20% of cells are positive; 2, 20–50% positive; 3, > 50% positive. The second parameter was the intensity of the immunostaining, which was scored as follows: 0, no staining; 1, weak staining; 2, moderate staining; 3, strong staining. Scores for the two parameters were multiplied, yielding a product with a value ranging from 0 to 9. Representative examples are shown in Supplementary Fig. 1.

### Immunofluorescence

Cells were fixed with 3% paraformaldehyde and permeabilized with 0.1% Triton X-100. The following primary antibodies and dilutions were used for immunofluorescence staining: anti-ZO-1 (1:100), anti-E-cadherin (1:200), anti-β-catenin (1:400), anti-RhoA (1:100), anti-laminin (1:200), and anti-giantin (1:200). F-actin was stained with rhodamine-phalloidin (Cat. R415; Invitrogen). Nuclei were stained with DAPI (4′,6-diamidino-2-phenylindole). Alexa Fluor 488 AffiniPure Donkey Anti-Mouse IgG antibody (H + L, Cat. 715-545-150) and Alexa Fluor 594 AffiniPure Donkey Anti-Rabbit IgG antibody (H + L Cat. 711-585-152) (Jackson ImmunoResearch, West Grove, PA, USA) were used as secondary antibodies. Images were obtained using an inverted fluorescence phase contrast microscope (BZ-X710, Keyence, Osaka, Japan).

### Image analysis

The relative staining intensity was measured using line scans of fluorescence intensities with ImageJ software^23^. Lines were drawn across randomly selected cell–cell junctions, and peak fluorescence intensities were measured using plot profile function. Colocalization was quantified by calculating Mander’s colocalization coefficient, using ImageJ Coloc 2 software^24^.

### Cell aggregation assay

Cells were seeded in 60-mm non-adherent dishes (Nunclon Sphera Dishes, Thermo Fisher Scientific) at a density of 20,000 cells/mL and cultured to form cell aggregates. After 10 days of culturing, the medium containing the cells was transferred into a 15-mL conical tube, and the cells were allowed to settle (pellet) by gravity sedimentation for 5 min to separate cell aggregates from cells that remained in suspension. The supernatant was recovered by careful aspiration, and the number (N1) of cells in the supernatant was counted. The pellets were dissociated into single cells by incubation with trypsin/EDTA followed by pipetting, and the number (N2) of cells in the pellets was counted. The percentage of aggregation was calculated as follows: % aggregation = [number of aggregated cells (N2)/(number of aggregated cells (N2) + number of singlet cells (N1)] × 100, using data from three independent experiments.

### RhoA activation assay

The RhoA activation assay was performed using the RhoA Pull-down Activation Assay Biochem Kit (Cat. #BK036; Cytoskeleton) according to the manufacturer's protocol. Total and active RhoA protein were quantified using ImageQuant TL software (GE Healthcare). The ratio of active to total RhoA was compared between *KAP3* wild-type (WT) and KO cells using densitometric data from three independent experiments.

### Cell viability assay

Cell viability was determined by the water-soluble tetrazolium salt assay (Cell Count Reagent SF, Nacalai Tesque, Kyoto, Japan), according to the manufacturer's protocol.

### Nocodazole treatment

Cells were treated by incubation under standard conditions for 16 h in culture medium supplemented with 1 µg/mL of nocodazole (Sigma-Aldrich, Tokyo, Japan).

### Statistical analysis

Statistical analyses were performed using SPSS Statistics (version 24.0; IBM, Armonk, NY, USA). Comparisons were made using a two-tailed Welch’s *t* test. The Kruskal–Wallis test was used to compare three groups. A *P* value of less than 0.05 was considered significant.

#### Ethics approval and consent to participate

This study received approval from the ethics committees of Kyoto Prefectural University of Medicine and was conducted in accordance with the Declaration of Helsinki.

## Results

### KAP3 expression is decreased in SRCC of the stomach

**Figure 1:**
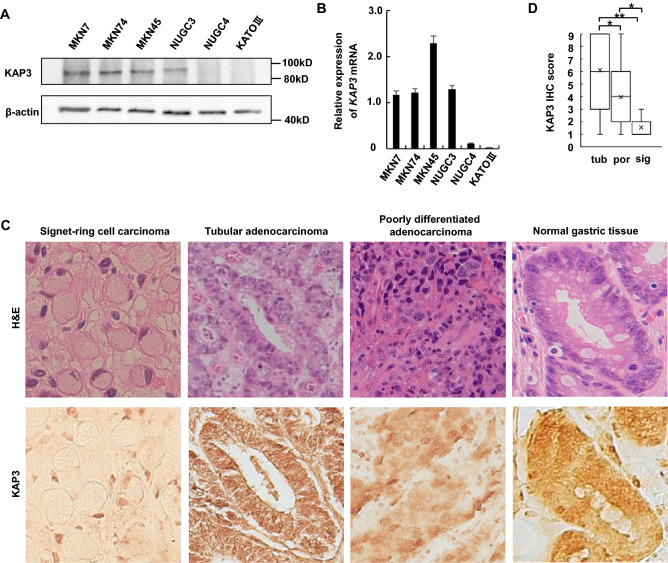
Loss of KAP3 expression in SRCC of the stomach. (**A**) Immunoblot analysis of KAP3 in six GC cell lines. β-actin was blotted as an internal (loading) control. (**B**) Levels of *KAP3* mRNA in the same GC cell lines, as determined by quantitative RT-PCR (n = 3). The expression level of *ACTB* was used as an endogenous control for the mRNA levels. Values are presented as mean ± SD. (**C**) Hematoxylin–eosin (H&E) staining and immunohistochemistry of KAP3 in three histological types of primary GC. Original magnification, ×200. (**D**) Semi-quantitative analysis of KAP3 expression in the three histological types of primary GC, which include tubular adenocarcinoma (tub; n = 21), poorly differentiated adenocarcinoma (por; n = 22), and signet-ring cell carcinoma (sig; n = 9). The middle line is median, upper and lower ends of box are 75th and 25th percentiles, and whiskers are maximum and minimum values. “X” indicates the mean value. *P < 0.05; **P < 0.01. *IHC* immunohistochemistry.

We investigated the gene expression prolife of GC using a web tool called Reference Expression dataset (RefEx; http://refex.dbcls.jp/)^25^ and noticed that *KAP3* mRNA expression, which is ubiquitous in mammalian cells, was markedly decreased in NUGC4 and KATOIII, cell lines that are derived from SRCC of the stomach. We confirmed that KAP3 expression, at both the protein and mRNA levels, was decreased in NUGC4 and KATOIII cells compared with cell lines derived from other histological subtypes of GC (i.e., tubular adenocarcinoma, MKN7 and MNK74 cells; poorly differentiated adenocarcinoma, MNK45 and NUGC3 cells), as determined by immunoblot and qRT-PCR analysis (Fig. 1A,B). Immunohistochemistry revealed that the expression levels of KAP3 were significantly lower in primary SRCC (sig) than in tubular adenocarcinoma (tub) and poorly differentiated adenocarcinoma (por) of the stomach (Fig. 1C,D).

### KAP3 depletion decreases cell–cell adhesion

**Figure 2:**
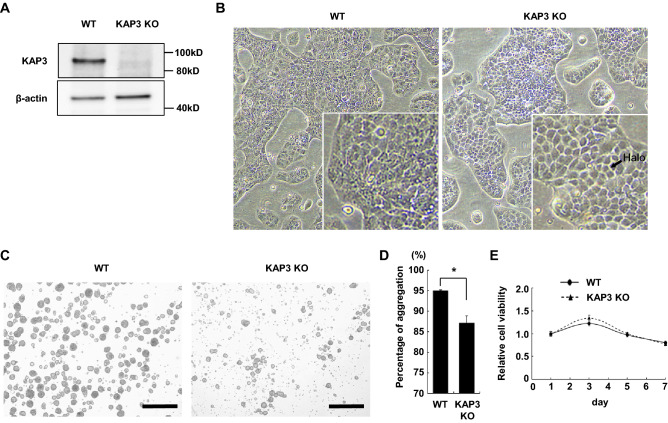
Morphological changes and decreased cell aggregation in *KAP3* KO cells. (**A**) Immunoblot analysis of KAP3 in *KAP3* wild-type (WT) and knockout (KO) cells derived from MNK74 cells. β-actin was blotted as an internal (loading) control. (**B**) Phase contrast images of *KAP3* WT and KO cells. The insets are magnified images of the respective micrographs. Original magnification, × 40. (**C,D**) Cell aggregation assay. Phase contrast images (C) and the percentage (D) of cell aggregation of *KAP3* WT and KO cells after 10 days of culturing in non-adherent dishes. Values are presented as mean ± SD. *P < 0.05. Original magnification, × 40; scale bars, 500 µm. (**E**) Cell viability assay of *KAP3* WT and KO cells cultured in non-adherent dishes (n = 5). Values are presented as mean ± SD.

To investigate the significance of KAP3 in the pathogenesis of SRCC, we established a *KAP3* knockout clone (*KAP3* KO) from MKN74 cells using CRISPR–Cas9-based gene editing. Depletion of KAP3 expression was confirmed in *KAP3* KO cells by immunoblotting analysis (Fig. 2A).

Phase contrast microscopy showed that *KAP3* KO cells, unlike WT cells, had a clear halo artifact^26^ around the cell boundaries (Fig. 2B), a finding suggestive of decreased (“looser”) cell–cell adhesion^27^.

To further evaluate cell–cell adhesion, we performed a cell aggregation assay on cells grown in non-adherent dishes. Cell aggregates were smaller in *KAP3* KO cells compared to WT cells (Fig. 2C), and the percentage of aggregated cells was significantly lower in the mutant than in the WT (Fig. 2D), consistent with the above-mentioned loosened cell–cell adhesion of *KAP3* KO cells.

To examine whether there was a difference in anoikis resistance between WT and *KAP3* KO cells, cell viability assays were performed in non-adherent 96-well plates. No difference in cell viability was observed between WT and *KAP3* KO cells (Fig. 2E), suggesting that the difference in cell aggregation was due to the difference in cell–cell adhesion, but not to anoikis resistance.

### KAP3 depletion causes RhoA inactivation and consequent failure of circumferential actomyosin cable formation

**Figure 3:**
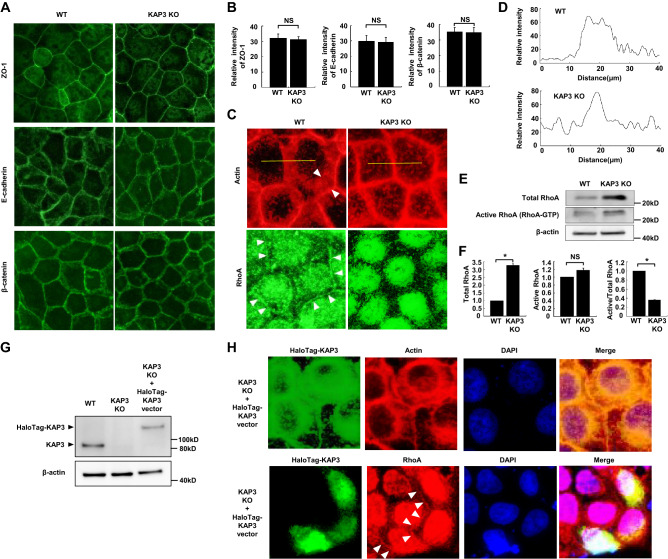
Inactivation of RhoA and impaired formation of a circumferential actomyosin cable in *KAP3* KO cells. (**A,B**) AJC components in *KAP3* WT and KO cells. Immunofluorescent staining (**A**) and the relative intensity (**B**) of ZO-1, E-cadherin, and β-catenin immunostaining across the cell junctions in *KAP3* WT and KO cells (n = 20). Values are presented as mean ± SD. (**C**) (*upper*) Immunofluorescence analysis of actin stained with rhodamine phalloidin in *KAP3* WT and KO cells. Arrowheads indicate circumferential actomyosin cables in WT cells. (*lower*) Immunofluorescence analysis of RhoA in *KAP3* WT and KO cells. Arrowheads indicate the localization of RhoA on the cell membrane in WT cells. RhoA signal in nuclei in WT and KO cells represents non-specific staining. Cells were viewed with an inverted fluorescence phase contrast microscope. (**D**) Quantification of immunosignals for F-actin across the cell junctions by densitometric scanning in WT and KO cells. The immunosignals were measured along the indicated yellow lines in (**C**; upper). (**E,F**) RhoA pull-down activation assay in *KAP3* WT and KO cells. (**E**) Levels of total and active (GTP-bound) RhoA detected by immunoblotting. β-actin was blotted as an internal (loading) control. (**F**) The relative expression of total and active RhoA, and the ratio of active to total RhoA, were calculated by densitometric analysis of immunoblots (n = 3). Values are presented as mean ± SD. (**G,H**) KAP3 rescue experiments using transient expression of HaloTag-KAP3 in *KAP3* KO cells. (**G**) Immunoblot analysis of HaloTag-KAP3 expression using anti-KAP3 antibody. (**H**) Immunofluorescence analysis of HaloTag-KAP3 (green), actin (*upper*; red), RhoA (*lower*; red), and DAPI (blue). HaloTag-KAP3 was detected by HaloTag ligand. Arrowheads indicate localization of RhoA on the membranes of cells that express HaloTag-KAP3.

Given the decreased cell–cell adhesion of *KAP3* KO cells, we postulated that AJC components might be altered in mutant cells. Although immunofluorescence for ZO-1, E-cadherin, and β-catenin showed no significant differences in fluorescence intensity and distribution between *KAP3* WT and KO cells (Fig. 3A,B), rhodamine-phalloidin staining indicated a different distribution of F-actin in the two cell lines (Fig. 3C, upper). WT cells exhibited dense staining of F-actin around the inside of the cell membrane, indicating the formation of circumferential actomyosin cables along the cell–cell junction. In contrast, *KAP3* KO cells exhibited a patchy distribution of F-actin in the cytoplasm, suggesting the failure of circumferential actomyosin cable formation. These findings were confirmed by quantification of immunosignals for F-actin across the cell junctions by densitometric scanning (Fig. 3D).

Because actomyosin contractility, which strengthens cell–cell adhesion, is regulated by active RhoA at the cell membrane, we examined expression of RhoA in WT and *KAP3* KO cells. Immunofluorescence analysis showed decreased localization of RhoA at the cell membrane in *KAP3* KO cells compared to WT cells (Fig. 3C, lower).

Another *KAP3* KO clone (KO #2), established independently, showed a similar phenotype for the subcellular mislocalization of F-actin and RhoA (Supplementary Fig. 2A, B). The SRCC cell line, NUGC4, completely lacks cell–cell adhesion (Supplementary Fig. 3A). We showed that NUGC4 cells also do not show circumferential actomyosin cable formation or the localization of RhoA at the cell membrane (Supplementary Fig. 3B).

RhoA activation was quantitated in *KAP3* WT and KO cells using a pull-down assay. The amount of total RhoA was significantly higher in KO cells than in WT cells (Fig. 3E,F), although there was no difference in the level of *RHOA* mRNA (data not shown) or in the amount of active RhoA (Fig. 3E,F) between the two cell lines. As a result, the ratio of active RhoA to total RhoA in *KAP3* KO cells was approximately three-fold lower than that in WT cells (Fig. 3E,F). These findings indicated that RhoA is inactivated in *KAP3* KO cells compared to WT cells.

To confirm these findings, we conducted genetic rescue experiments by introducing a construct encoding HaloTag-fused KAP3 (HaloTag-KAP3) into *KAP3* KO cells (Fig. 3G). The dense staining of F-actin around the inside of the cell membrane was restored in cells expressing HaloTag-KAP3 (Fig. 3H, upper). Furthermore, RhoA was re-localized to the cell membrane in cells that expressed HaloTag-KAP3 (Fig. 3H, lower).

### KAP3 depletion inhibits post-Golgi transport of laminin

**Figure 4:**
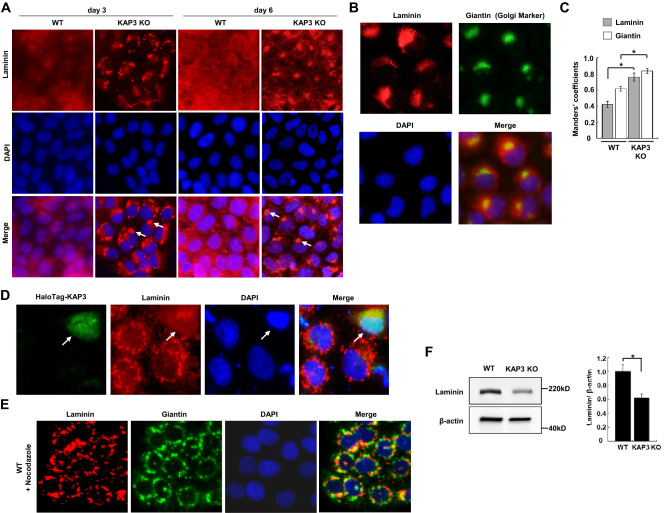
Impairment of post-Golgi transport of laminin and decreased laminin expression in *KAP3* KO cells. (**A**) Immunofluorescent staining of laminin (red) in *KAP3* WT and KO cells at Day 3 or Day 6 after passage. Nuclei were counterstained with DAPI (blue). Arrows indicate an accumulation of laminin in *KAP3* KO cells. (**B**) Co-localization of laminin and giantin (a Golgi marker) in *KAP3* KO cells. *KAP3* KO cells were stained with anti-laminin antibody (red), anti-giantin antibody (green), and DAPI (blue), and were viewed with an inverted fluorescence phase contrast microscope. (**C**) Manders’ colocalization coefficient of laminin and giantin in *KAP3* WT and KO cells (n = 50). Values are presented as mean ± SD. *P < 0.05. (**D**) Immunofluorescence analysis of *KAP3* KO cells transfected with the HaloTag-KAP3-encoding vector. Cells were stained with HaloTag ligand (green), anti-laminin antibody (red), and DAPI (blue) at Day 3 after transfection. Arrows indicate cells expressing HaloTag-KAP3. (**E**) Immunofluorescence analysis of *KAP3* WT cells that were treated with 1 µg/mL of nocodazole for 16 h. Cells were labeled as in (**B**). (**F**) Immunoblot analysis of laminin in *KAP3* WT and KO cells. β-actin was blotted as an internal (loading) control. Values are presented as mean ± SD. (n = 3). *P < 0.05.

Because the inactivation of RhoA also decreases microtubule stability, and microtubule disruption causes basement membrane breakdown^28^, we investigated expression of laminin, a key component of the basement membrane, in WT and *KAP3* KO cells. Immunofluorescence analysis revealed an accumulation of laminin, which stained as spots with a small, rounded appearance or crescent shape, adjacent to the nucleus in *KAP3* KO cells; in contrast, laminin was uniformly distributed throughout the cytoplasm in WT cells (Fig. 4A). Double staining of laminin and giantin, a Golgi marker, indicated the localization of laminin primarily in the peri-Golgi area in *KAP3* KO cells (Fig. 4B). Mander’s colocalization coefficient analysis confirmed that the fraction of laminin colocalized with giantin was significantly higher in KO cells than in WT cells (Fig. 4C). Furthermore, the rescue experiments showed that laminin was re-distributed uniformly throughout the cytoplasm in cells that expressed HaloTag-KAP3 (Fig. 4D). Another *KAP3* KO clone (KO #2) also showed the colocalization of laminin with giantin (Supplementary Fig. 2C). These findings indicated pooling of laminin in the peri-Golgi area in *KAP3* KO cells, suggesting inhibition of the post-Golgi transport of laminin in the mutant cell lines.

In the SRCC cell line NUGC4, a portion of the laminin appeared to co-localize with giantin (Supplementary Fig. 3C). Rescue experiments in NUGC4 cells suggested that laminin is distributed more uniformly in the cytoplasm in *KAP3* KO cells that express HaloTag-KAP3, compared to *KAP3* KO cells that do not express the fusion protein (Supplementary Fig. 3D).

WT cells were treated with nocodazole, an inhibitor of microtubule polymerization. The treatment resulted in co-localization of laminin with giantin (Fig. 4E), suggesting that the post-Golgi transport of laminin depends on microtubules.

Moreover, the expression of laminin protein was decreased in *KAP3* KO cells compared to WT cells (Fig. 4F). Expression of laminin also was significantly decreased in two SRCC cell lines, NUGC4 and KATOIII, compared with MKN74 cells (i.e., *KAP3* WT cells), the tubular adenocarcinoma cell line (Supplementary Fig. 3E).

### Laminin deposition at basement membrane is impaired in SRCC of the stomach

**Figure 5:**
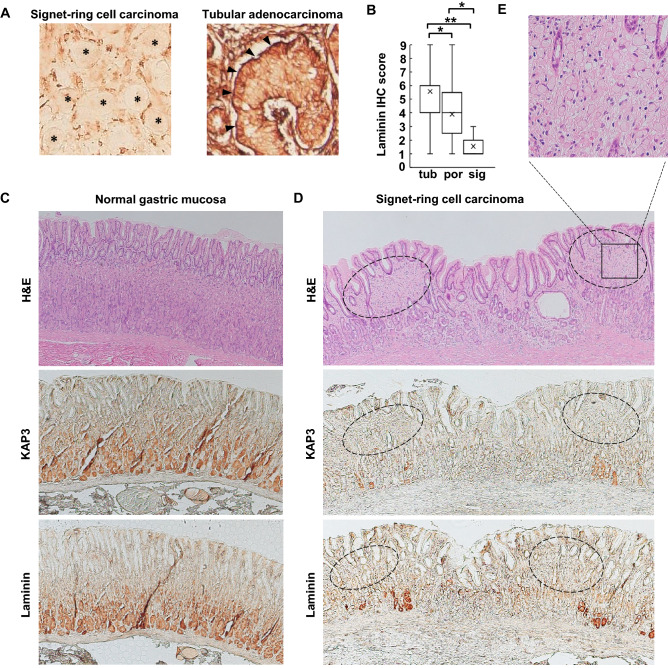
Impairment of laminin deposition at basement membrane with loss of *KAP3* expression in SRCC. (**A**) Immunohistochemistry of laminin in clinical samples of SRCC and tubular adenocarcinoma of the stomach. Arrowheads indicate laminin at basement membranes. Asterisks indicate cells of SRCC. Staining seen in SRCC is interstitial tissue. Original magnification, ×200. (**B**) Semi-quantitative analysis of laminin expression in the three histological types of primary GC, which include tubular adenocarcinoma (tub; n = 21), poorly differentiated adenocarcinoma (por; n = 22), and signet-ring cell carcinoma (sig; n = 9). The middle line is median, upper and lower ends of box are 75th and 25th percentiles, and whiskers are maximum and minimum values. “X” indicates the mean value. *P < 0.05; **P < 0.01. *IHC* immunohistochemistry. (**C–E**) H&E and immunohistochemical staining of KAP3 and laminin in the normal gastric mucosa (**C**) and in an early gastric cancer of SRCC limited to the mucosa (**D**). Dotted circles indicate foci of SRCC. Original magnification, ×40. (**E**) Magnified view of the square area in the H&E-stained section of SRCC shown in (**D**). Note that separate (non-adjacent) sections were used for H&E and immunohistochemical staining of KAP3 and laminin (because the original specimens were sectioned multiple times for use in various pathological diagnoses and studies).

Based on the finding that the post-Golgi transport of laminin is inhibited in *KAP3* KO cells, we postulated that laminin secretion might be reduced, leading to a defect of the basement membrane, in SRCC lacking KAP3 expression. To test this hypothesis, we performed immunohistochemistry for laminin in specimens of primary GC. Laminin was present at the basement membrane on which tubular adenocarcinoma cells resided, judging from the sheet-like deposition of stained material at the basal surface of those cells (Fig. 5A). However, laminin deposition was not observed at the surface of SRCC cells (Fig. 5A). Additionally, cytoplasmic staining of laminin in SRCC cells was weaker than that in tubular adenocarcinoma cells. Expression levels of laminin were significantly decreased in primary SRCC (sig) compared to tubular adenocarcinoma (tub) and poorly differentiated adenocarcinoma (por) of the stomach (Fig. 5B).

We further examined expression of KAP3 and laminin by immunohistochemistry in the normal gastric mucosa and in an early GC composed of a pure histological type of SRCC that is limited to the mucosa (Fig. 5C–E). KAP3 and laminin were co-expressed in the normal gastric mucosa (Fig. 5C). The staining intensities of KAP3 and laminin at the base of gastric glands were stronger than in the superficial portion. However, the expression of KAP3 and laminin was absent in SRCC (Fig. 5D, shown in dotted circles). Intriguingly, expression of KAP3 and laminin also was sparse or absent in non-tumor cells that were immediately adjacent to SRCC (Fig. 5D).

Taken together, these findings suggested that loss of KAP3 may impair the formation of the basement membrane through inhibition of the post-Golgi transport of laminin in SRCC of the stomach, and that expression of KAP3 and laminin may be decreased in the background gastric mucosa of SRCC.

## Discussion

In the present study, we observed decreased expression of KAP3 in SRCC of the stomach. KAP3 is a component of KIF3, a multi-subunit protein that is ubiquitously expressed in mammalian cells^29^. KIF3 regulates microtubule-based transport that is critical for membrane organelle transport, anterograde fast axonal transport, and early embryonic and neuronal development^30–34^; KIF3 also regulates microtubule organization^35^, as well as intraflagellar transport that is indispensable for the formation and maintenance of cilia and flagella^36^. Dysregulation of KIF3 contributes to ciliopathies such as polycystic kidney disease^37^, retinitis pigmentosa^38^, situs inversus^39^, and schizophrenia^40^. KIF3 also has been implicated in the tumorigenesis of several types of cancer, including brain tumor^32^, medulloblastoma^41^, breast cancer^42^, non-small cell lung cancer^43^, and prostate cancer^44^, primarily via dysregulation of Wnt signaling and ciliary function. KIF3A restrains canonical Wnt signaling through ciliary and non-ciliary mechanisms^32,45^. KIF3 regulates cell migration by transporting the tumor suppressor adenomatous polyposis coli (APC) to membrane protrusions^46^. KAP3 is known to interact with APC^46^, small GTP-binding protein GDP dissociation stimulator (Smg GDS)^47^, PAR-3^48^, and fodrin^49^. KAP3 deficiency in mouse neuroepithelium leads to malignant transformation due to impaired post-Golgi transport of N-cadherin^32^, suggesting a potential tumor-suppressing activity for KAP3. Although the mechanism by which KAP3 expression is decreased in SRCC of the stomach is unknown, we hypothesize that loss of KAP3 may contribute to the development and progression of SRCC.

Our results suggested that loss of KAP3 decreased the localization of RhoA at the cell membrane and consequently reduced RhoA activity. The inactivation of RhoA caused the failure of circumferential actomyosin cable formation, leading to decreased cell–cell adhesion in *KAP3* KO cells. Although the mechanism by which KAP3 depletion led to impaired localization of RhoA at the cell membrane was not elucidated, Smg GDS may be involved in the mechanism. Smg GDS interacts with small GTPases possessing a C-terminal polybasic region (PBR), including proteins such as Rap1, RhoA, Rac1, and K-Ras. The PBR controls various functions of these small GTPases, including their interaction with other proteins and association with membranes^50^. Smg GDS is known to have direct interactions with both KAP3 and RhoA^47^. In addition to its function as guanine-nucleotide exchange factor (GEF) that activates RhoA, Smg GDS acts as a chaperone controlling RhoA prenylation, a modification that is essential for membrane localization^51^. KAP3 depletion might alter the localization and functions of Smg GDS, thereby inhibiting RhoA activity and localization at the cell membrane.

Interestingly, our findings showed that RhoA accumulated to higher levels in *KAP3* KO cells compared to WT cells. Most of the RhoA protein in the cell is maintained in an inactive form and protected from degradation by cytosolic chaperones, including Rho-specific guanine nucleotide dissociation inhibitors (RhoGDIs); only a small fraction of all RhoA protein is activated, primarily at the plasma membrane^52^. Activated RhoA at cellular protrusions is targeted for degradation by E3 ubiquitin ligases such as Smurf-1^53^. Therefore, the total levels of RhoA protein are determined primarily by the amount bound to RhoGDIs in the cell^52^. Given these previous findings, impaired RhoA localization at the cell membrane as a result of KAP3 depletion may increase the amount bound to cytosolic RhoGDIs, leading to the observed increase in total RhoA protein.

*RHOA* mutations recurrently occur in non-SRCC diffuse-type GC (i.e., poorly differentiated adenocarcinoma). Several lines of evidence have indicated that mutant RhoA works in a gain-of-function manner^54^. In contrast, *RHOA* mutation is rare in SRCC^14^. Our findings showed that loss of KAP3, which occurs in SRCC, causes RhoA inactivation. The clinicopathological significance of altered RhoA signaling may differ between SRCC and non-SRCC diffuse-type GC.

The post-Golgi transport of laminin was inhibited in *KAP3* KO cells. ECM proteins, including laminin, are synthesized on ribosomes bound to the endoplasmic reticulum (ER) membrane, post-translationally modified in the ER, transported through the Golgi complex in vesicles, and then secreted to the cell surface. KAP3 is known to be concentrated around the ER^47^, implicating KAP3 in the secretory pathway. While one previous study showed the involvement of KAP3 in shuttle transport between the ER and the Golgi^55^, another demonstrated the contribution of KAP3 to the post-Golgi transport of N-cadherin to the cell-surface^32^. Thus, the precise function of KAP3 in intracellular transport remains elusive.

Teng et al.^32^ have shown that post-Golgi transport of N-cadherin by the KIF3 molecular motor complex is crucial for maintaining a balance between proliferation and cell–cell adhesion of neural progenitor cells, and that the subcellular localization of N-cadherin is disrupted and cell aggregation activity is decreased in KAP3-deficient cells.

Although the mechanism by which KAP3 depletion impaired the post-Golgi transport of laminin still needs to be elucidated, this effect may be mediated in part by the inactivation of RhoA. Notably, RhoA and its effector mDia1 regulate the stabilization of microtubules^56^, which are used as tracks for fast and directed transport, and the RhoA–mDia1 pathway is involved in regulation of the Golgi structure^57^.

The in vitro findings that the post-Golgi transport of laminin was inhibited in *KAP3* KO cells were supported by immunohistochemical analysis using clinical specimens of GC. While laminin was present at the basement membrane of tubular adenocarcinoma cells, this protein was not observed at the surface of SRCC cells, suggesting the impairment of basement membrane formation in SRCC. Microscopically, SRCC exhibits discohesive cells infiltrating the lamina propria even in its early stage^58^. One possible explanation for this observation is a defect of the basement membrane that separates epithelial cell sheets from the lamina propria; in normal tissues, the basement membrane acts as a mechanical barrier that prevents malignant cells from invading the deeper tissues.

In the specimens of early SRCC, expression of KAP3 and laminin was absent in tumor cells. Notably, not only tumor cells themselves but also immediately adjacent non-tumor cells showed decreased expression of KAP3. Although the mechanism and significance of this finding are unknown, we hypothesize that the decreased expression of KAP3 may occur in the background gastric mucosa of SRCC prior to the development of SRCC, and so may serve as an early marker of carcinogenesis in SRCC.

Given that the gastric epithelium is continually exposed to the harsh luminal environment, discohesive single cells of SRCC should readily be cleared from the epithelium unless such cells are somehow retained in the lamina propria. Several studies have suggested that the development of discohesive gastric cells into cancer is difficult^59,60^. According to Hayakawa et al*.*^60^, conditional knockout of *CDH1* in mouse gastric isthmus stem cells results in the development of atypical cells similar to SRCC. However, these atypical cells are gradually depleted before subsequently disappearing, and are incapable of developing into SRCC. Since *CDH1* and *RHOA* mutations often co-occur in diffuse-type GC, most studies have assumed that anoikis inhibition by an altered RhoA pathway enables discohesive gastric cells to develop into SRCC^60,61^. Nonetheless, based on our observation, we assume that changes in the basement membrane resulting from the loss of KAP3 enable tumor cells to easily invade the lamina propria and thereby facilitate the initiation of SRCC. KAP3 deficiency decreased cell–cell adhesion, and impaired basement membrane formation. These traits may enable tumor cells to deviate from epithelial cell sheets as single cells, and to persist in the lamina propria, leading to the initiation and progression of SRCC.

In conclusion, loss of KAP3 was specifically detected in SRCC of the stomach, and was shown to result in decreased RhoA-mediated cell–cell adhesion and impaired basement membrane formation through inhibition of the post-Golgi transport of laminin. This study provides new insights into the carcinogenesis of SRCC by uncovering new functions of KAP3 in the regulation of RhoA signaling and laminin transport. The precise molecular and physical mechanisms underlying KAP3 function in these processes are challenging subjects for future study. Loss of KAP3 expression could be a useful marker that predicts onset risk of SRCC, and could serve as a molecular target for treatment of this cancer.

## Supplementary Information

Supplementary Figures.

## Data Availability

The datasets used and/or analyzed in the current study are available from the corresponding author on reasonable request.
