# Supplementary material for: Loss of KAP3 decreases intercellular adhesion and impairs intracellular transport of laminin in signet ring cell carcinoma of the stomach
Source: Sci Rep. 2022 Mar 23;12:5050. doi: 10.1038/s41598-022-08904-8 (PMC8943207; doi:10.1038/s41598-022-08904-8)
Supplement: Supplementary file 1 — Supplementary Figures. [file 41598_2022_8904_MOESM1_ESM.pdf]

Supplementary Information

Loss of KAP3 decreases intercellular adhesion and impairs intracellular transport of laminin in signet ring cell carcinoma of the stomach

Tomohiro Soda[1], Yasuyuki Gen[1], Kei Terasaki[1], Naoto Iwai[1], Tomoko Kitaichi[1], Osamu Dohi[1], Hiroyoshi Taketani[1], Yuya Seko[1], Atsushi Umemura[1], Taichiro Nishikawa[1], Kanji Yamaguchi[1], Michihisa Moriguchi[1], Hideyuki Konishi[1], Yuji Naito[1], Yoshito Itoh[1], and Kohichiroh Yasui[2] *

[1] Department of Molecular Gastroenterology and Hepatology, Kyoto Prefectural University of Medicine, Kamigyo, Kyoto 602-8566, Japan

[2] School of Health Sciences, Bukkyo University, Nakagyo, Kyoto 604-8418, Japan

***Correspondence to:** Kohichiroh Yasui MD, PhD
School of Health Sciences, Bukkyo University, Nakagyo, Kyoto 604-8418, Japan
Tel: +81-75-491-2141; Fax: +81-75-366-5757
E-mail: k-yasui@bukkyo-u.ac.jp

This PDF file includes:

Supplementary Figures. S1 to S3 and Uncropped Western Blots.

Supplementary Figure S1

A

Stained tumor cell	Propotion score (PS)	Stainig Intensity	Intensity score (IS)
0%	0	Negative	0
＜20%	1	Weak	1
20-50%	2	Moderate	2
＞50%	3	Strong	3

B

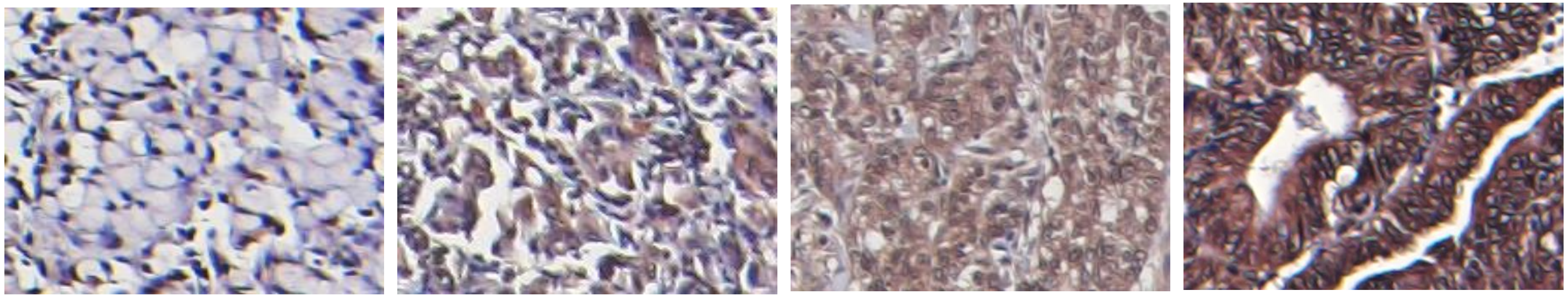

Score = 1 Score = 4 Score = 6 Score = 9

Supplementary Figure S1. Semi-quantitative tissue scoring of KAP3 and laminin in primary GC tissues. **(A)** Scoring criteria. **(B)** Representative cases of KAP3 immunohistochemistry scores.

Supplementary Figure S2

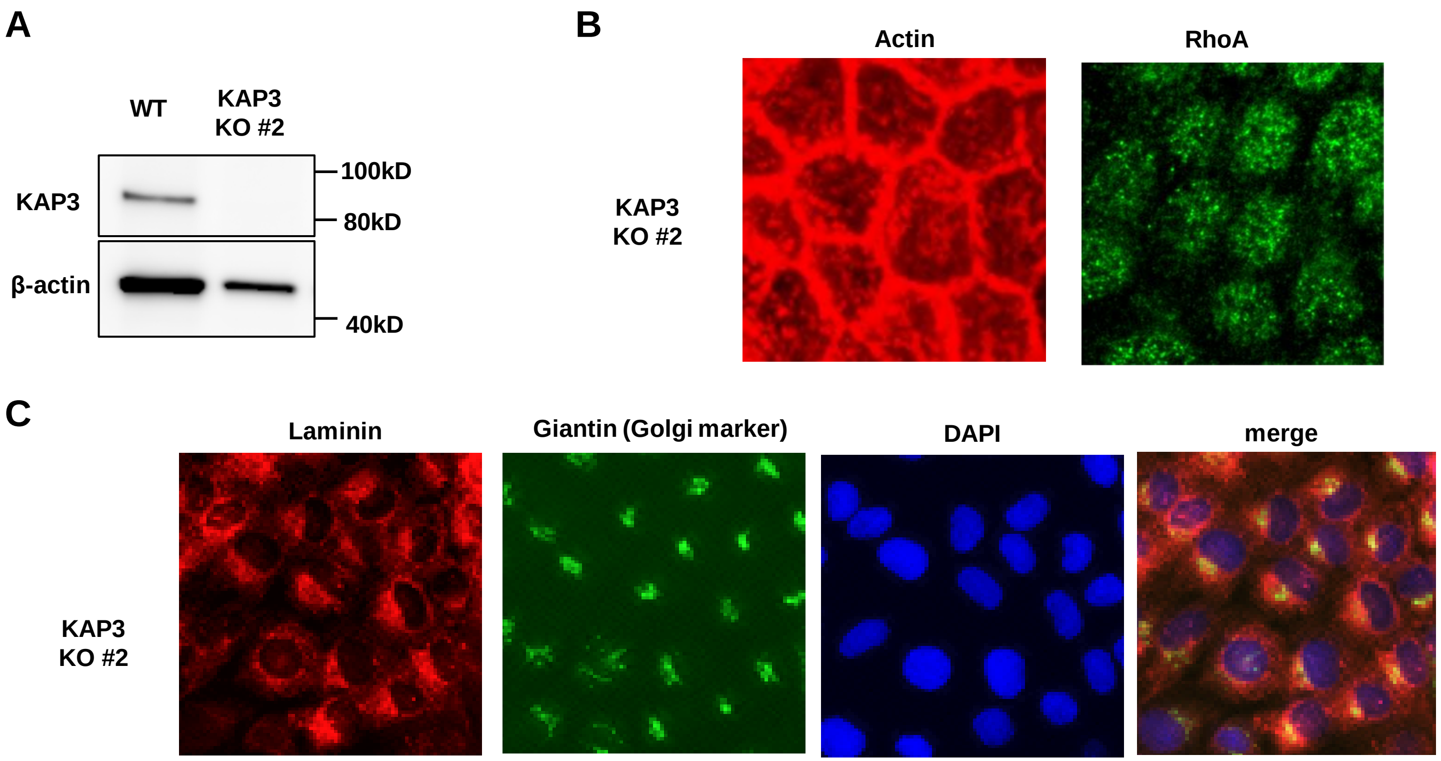

Supplementary Figure S2. Subcellular localization of actin, RhoA, and laminin in *KAP3* knockout (KO) #2 cells.
(A) Immunoblot analysis of KAP3 in *KAP3* wild-type (WT) and KO #2 cells. β-actin was blotted as an internal (loading) control. **(B)** Immunofluorescence analysis of actin and RhoA in *KAP3* KO #2 cells. RhoA signal in cell nuclei represents non-specific staining. **(C)** Co-localization of laminin and giantin (a Golgi marker) in *KAP3* KO #2 cells. The cells were stained with anti-laminin antibody (red), anti-giantin antibody (green), and DAPI (blue).

Supplementary Figure S3

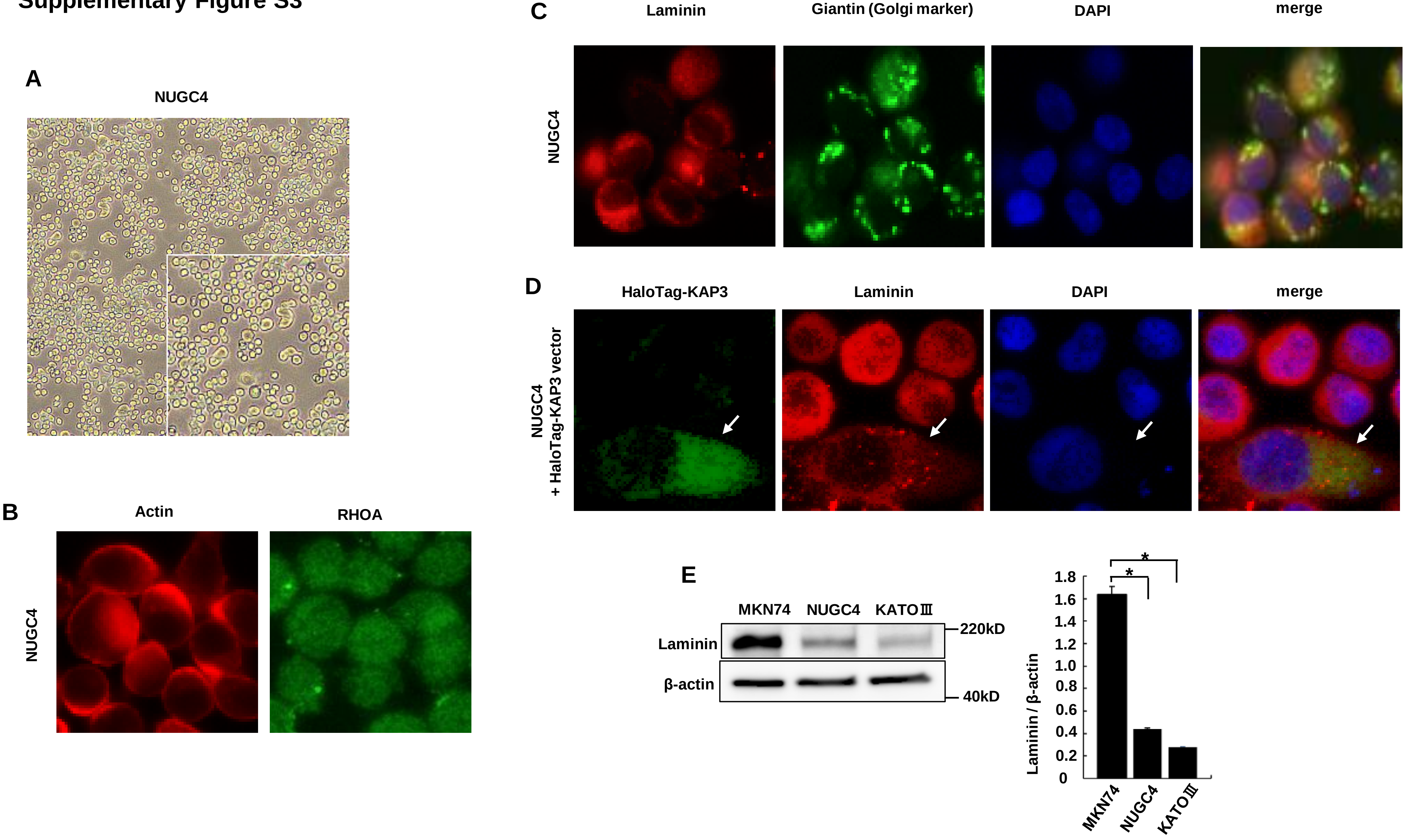

Supplementary Figure S3. Impaired cell-cell adhesion and post-Golgi transport of laminin in SRCC cell lines.

(A) Phase contrast images of a SRCC cell line, NUGC4. Original magnification, × 40. **(B)** Immunofluorescent staining of actin and RhoA in NUGC4 cells. **(C)** Co-localization of laminin and giantin (a Golgi marker) in NUGC4 cells. Cells were stained with anti-laminin antibody (red), anti-giantin antibody (green), and DAPI (blue). **(D)** Immunofluorescence analysis of NUGC4 cells transfected with the HaloTag-KAP3-encoding vector. Cells were stained with HaloTag ligand (green), anti-laminin antibody (red), and DAPI (blue). Arrows indicate cells expressing HaloTag-KAP3. **(E)** Immunoblot analysis of laminin in MKN74 cells and two SRCC cell lines (NUGC4 and KATO-III). β-actin was blotted as an internal (loading) control. Values are presented as mean ± SD. (n = 3). $*P < 0.05$.

Uncropped Western Blots

Figure 1A

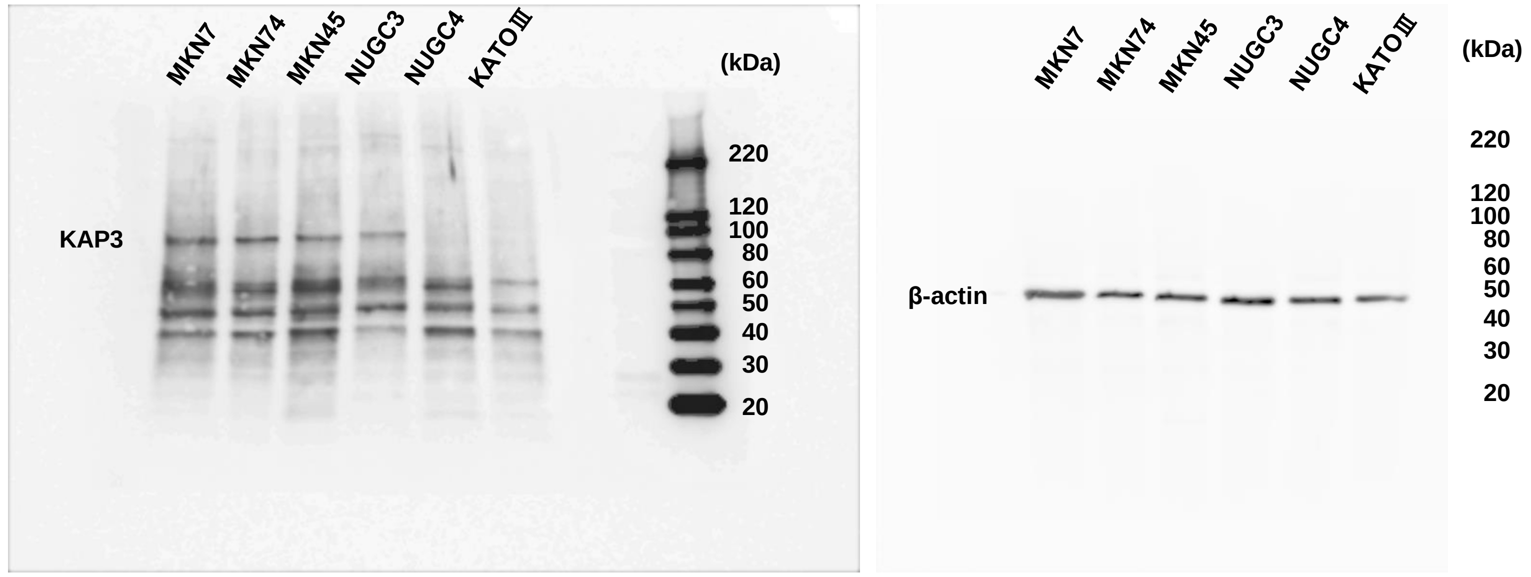

Figure 2A

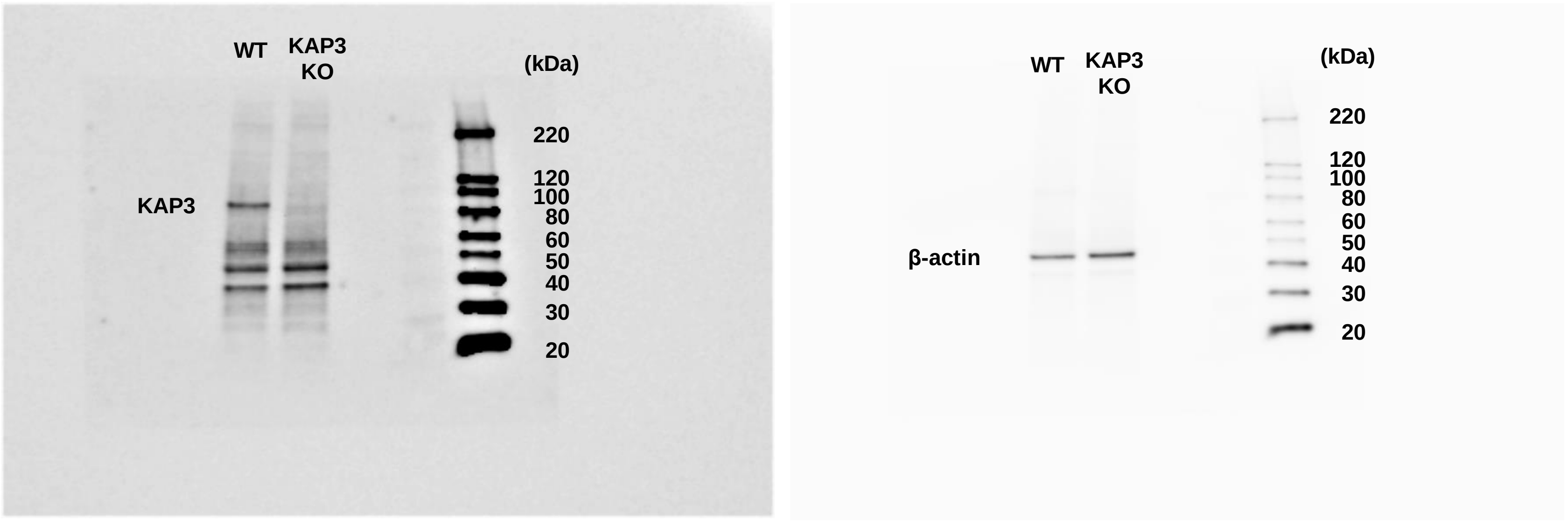

Uncropped Western Blots

Figure 3E

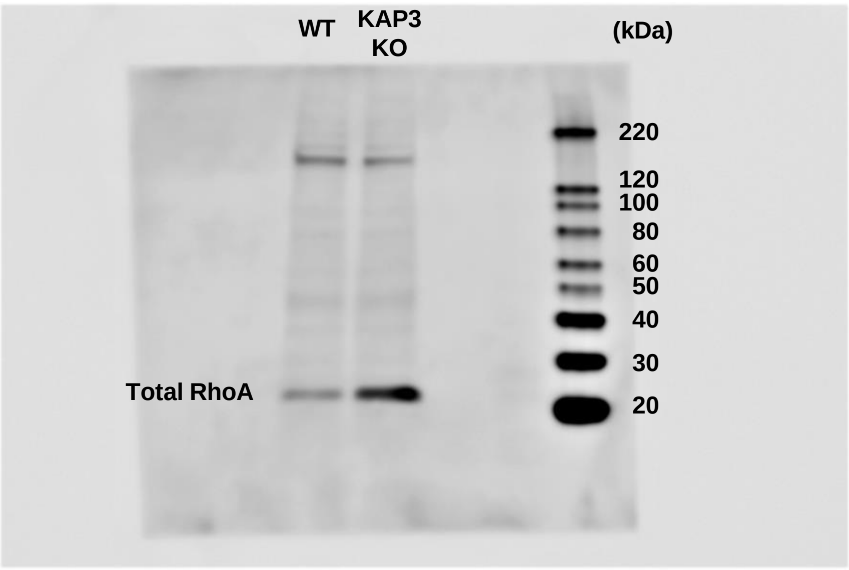

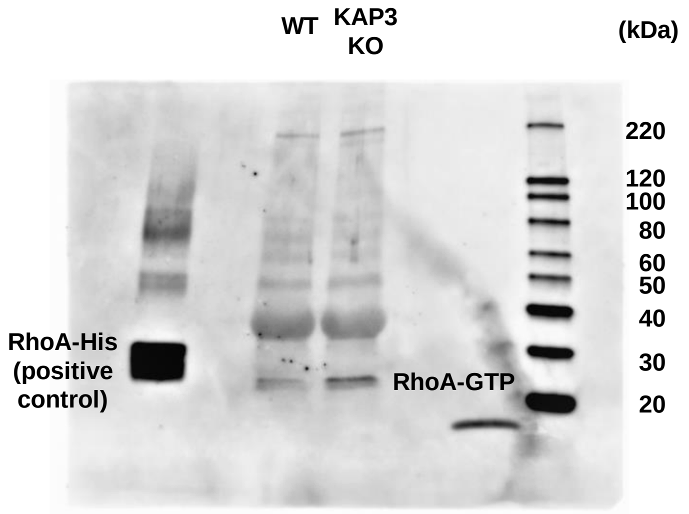

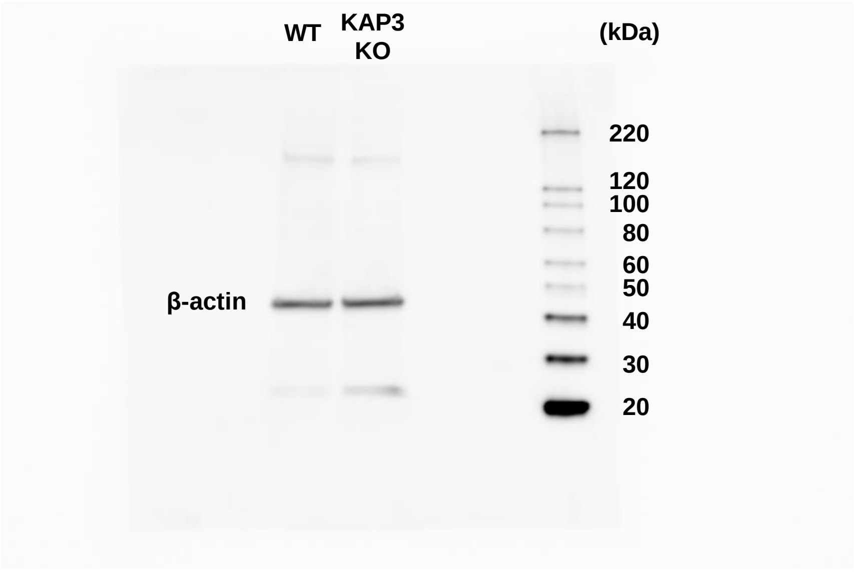

Figure 3G

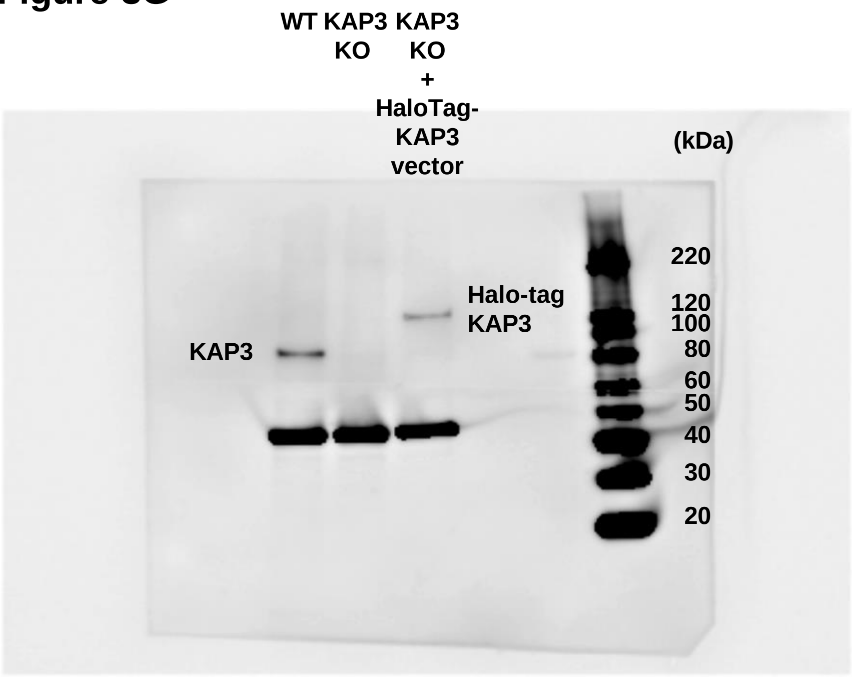

The membrane was cut into upper and lower sections for the separate detection of KAP3 and loading control (β-actin), respectively. Long exposure.

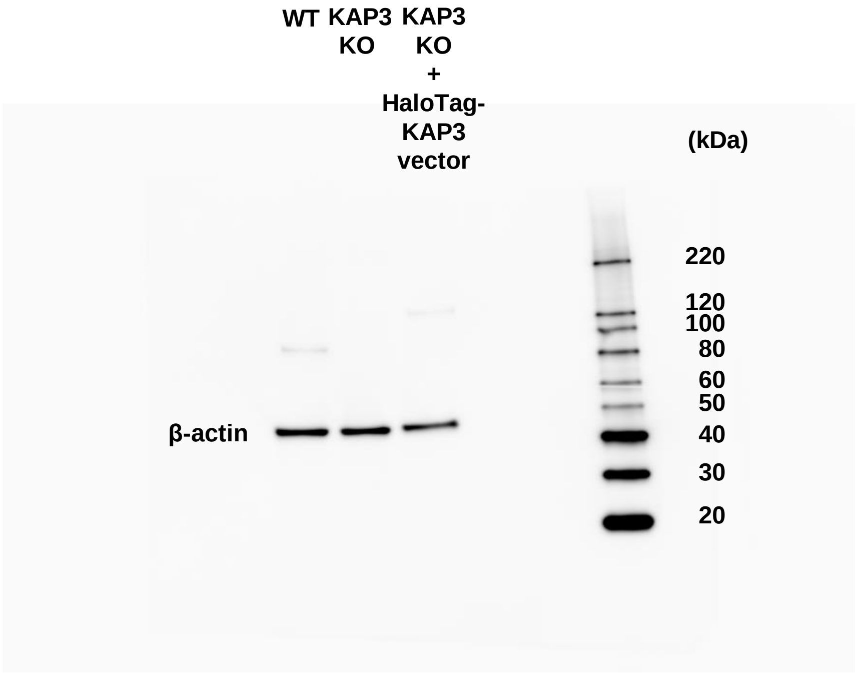

The membrane was cut into upper and lower sections for the separate detection of KAP3 and loading control (β-actin), respectively. Short exposure.

Uncropped Western Blots

Figure 4F

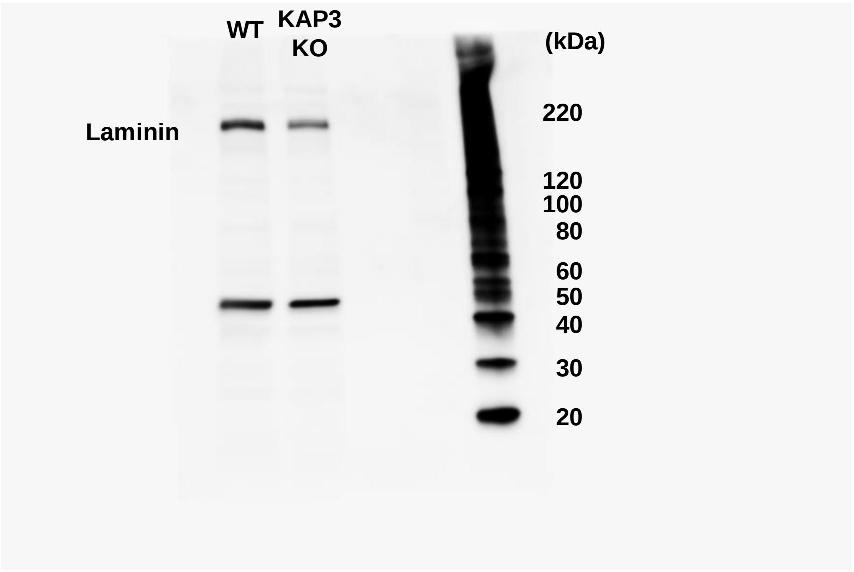

The membrane was cut into upper and lower sections for the separate detection of laminin and loading control (β-actin), respectively. Long exposure.

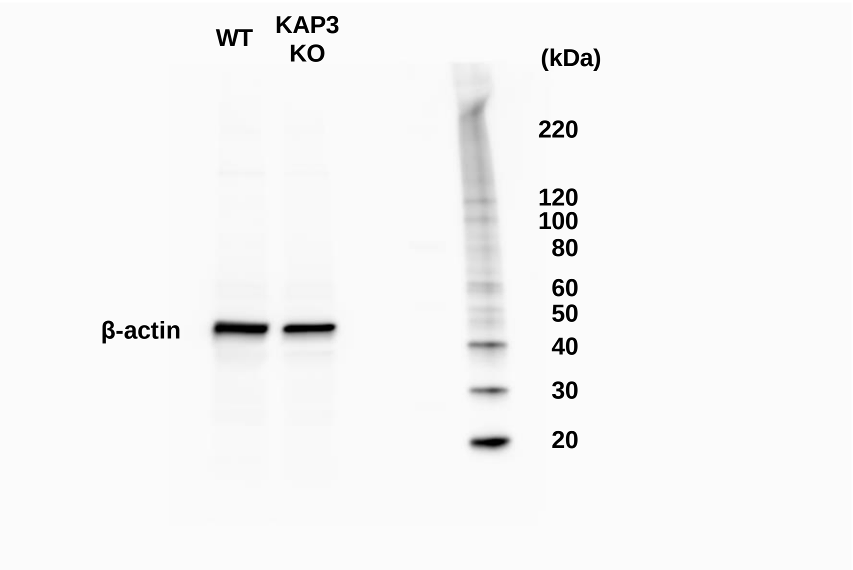

The membrane was cut into upper and lower sections for the separate detection of laminin and loading control (β-actin), respectively. Short exposure.

Uncropped Western Blots

Supplementary Figure S2A

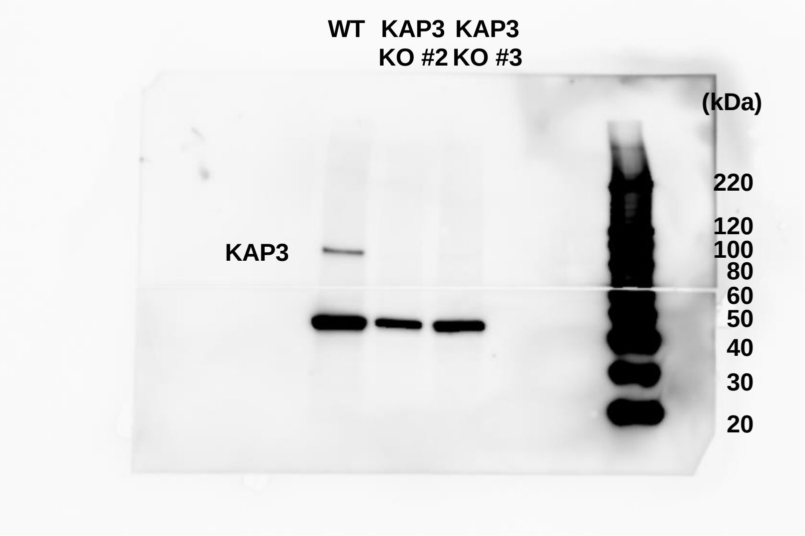

The membrane was cut into upper and lower sections for the separate detection of KAP3 and loading control (β-actin), respectively. Long exposure.

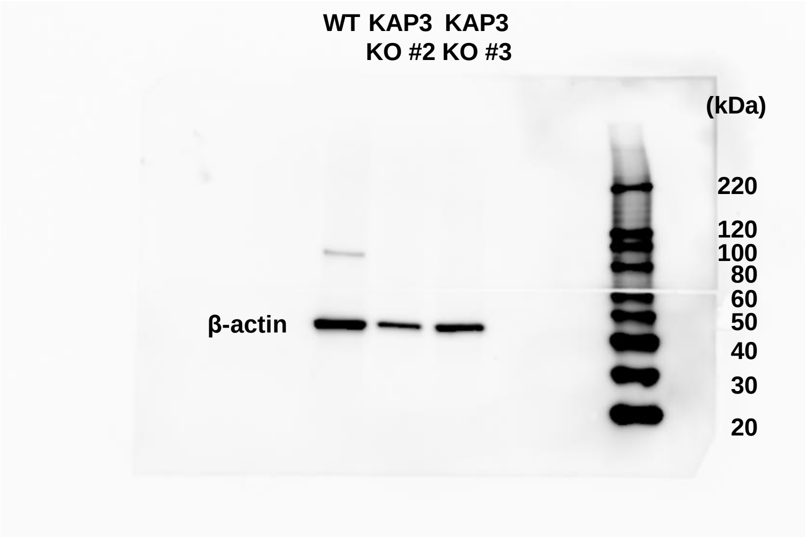

The membrane was cut into upper and lower sections for the separate detection of KAP3 and loading control (β-actin), respectively. Short exposure.

Supplementary Figure S3E

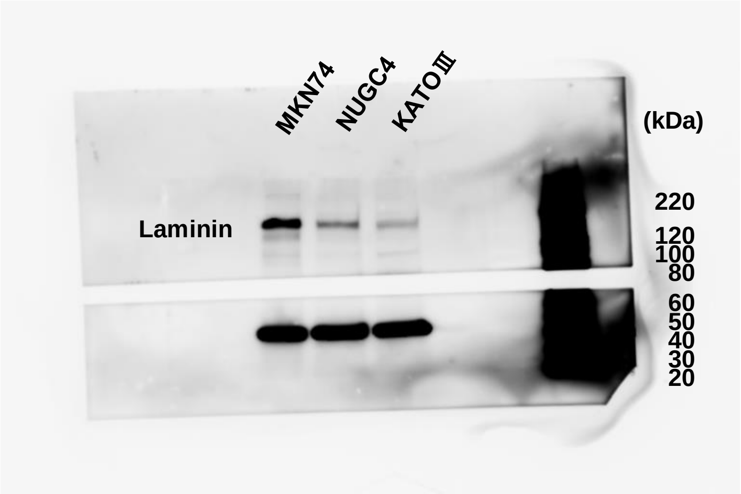

The membrane was cut into upper and lower sections for the separate detection of laminin and loading control (β-actin), respectively. Long exposure.

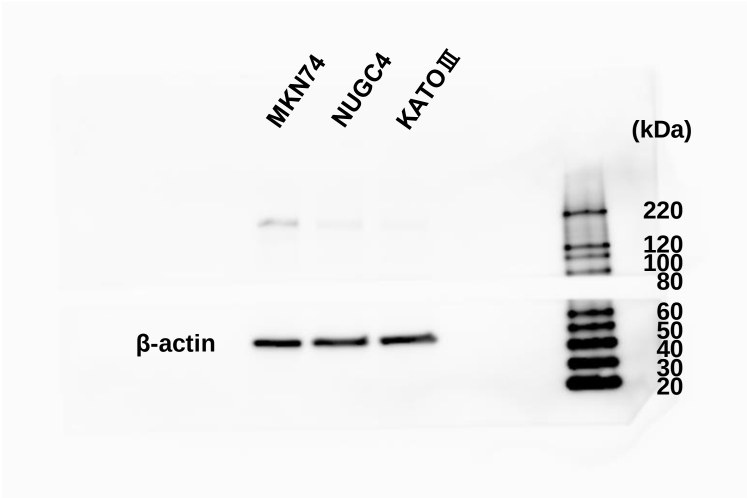

The membrane was cut into upper and lower sections for the separate detection of laminin and loading control (β-actin), respectively. Short exposure.
